# Molecular basis of the persistence of chloramphenicol resistance among *Escherichia coli* and *Salmonella* spp. from pigs, pork and humans in Thailand

**DOI:** 10.1371/journal.pone.0304250

**Published:** 2024-05-24

**Authors:** Jiratchaya Puangseree, Rangsiya Prathan, Songsak Srisanga, Rungtip Chuanchuen

**Affiliations:** 1 Research Unit in Microbial Food Safety and Antimicrobial Resistance, Department of Veterinary Public Health, Faculty of Veterinary Science, Chulalongkorn University, Bangkok, Thailand; 2 Center for Antimicrobial Resistance Monitoring in Food-borne Pathogens, Faculty of Veterinary Science, Chulalongkorn University, Bangkok, Thailand; North Carolina State University, UNITED STATES

## Abstract

This study aimed to investigate the potential mechanisms associated with the persistence of chloramphenicol (CHP) resistance in *Escherichia coli* and *Salmonella enterica* isolated from pigs, pork, and humans in Thailand. The CHP-resistant *E*. *coli* (n = 106) and *Salmonella* (n = 57) isolates were tested for their CHP susceptibility in the presence and absence of phenylalanine arginine β-naphthylamide (PAβN). The potential co-selection of CHP resistance was investigated through conjugation experiments. Whole genome sequencing (WGS) was performed to analyze the *E*. *coli* (E329, E333, and E290) and *Salmonella* (SA448, SA461, and SA515) isolates with high CHP MIC (32–256 μg/mL) and predominant plasmid replicon types. The presence of PAβN significantly reduced the CHP MICs (≥4-fold) in most *E*. *coli* (67.9%) and *Salmonella* (64.9%). Ampicillin, tetracycline, and streptomycin co-selected for CHP-resistant *Salmonella* and *E*. *coli*-transconjugants carrying *cmlA*. IncF plasmids were mostly detected in *cmlA* carrying *Salmonella* (IncFIIAs) and *E*. *coli* (IncFIB and IncF) transconjugants. The WGS analysis revealed that class1 integrons with *cmlA1* gene cassette flanked by IS*26* and Tn*As1* were located on IncX1 plasmid, IncFIA(HI1)/HI1B plasmids and IncFII/FIB plasmids. IncFIA(HI1)/HI1B/Q1in SA448 contained *catA* flanked by IS*1B* and Tn*As3*. In conclusion, cross resistance through proton motive force-dependent mechanisms and co-selection by other antimicrobial agents involved the persistence of CHP-resistance in *E*. *coli* in this collection. Dissemination of CHP-resistance genes was potentially facilitated by mobilization via mobile genetic elements.

## Introduction

One of the primary drivers to the emergence and spread of antimicrobial resistance (AMR), a serious global public health threat, is the indiscriminate use of antimicrobials (AMU) [1]. AMR develops more rapidly through the inappropriate and excessive use of antimicrobial agents and therefore, reducing or ceasing the use of antimicrobials is expected to lessen the likelihood of AMR bacteria development and distribution [2]. In other words, AMR would have vanished if the AMU had been removed.

In contrast, bacteria resistant to restricted antimicrobial drugs have been consistently isolated. For example, the use of chloramphenicol in food animals has been banned in many countries e.g., the US, Canada, Australia, Japan and China since 1994 due to being a cause of aplastic anemia [3]. The antibiotic has been outlawed in Thailand since 1998 [4]; nonetheless, chloramphenicol-resistant bacteria are continuously isolated from food animals and meat e.g., pig [5], poultry [6], pork and chicken [7, 8]. It was suggested to be the result of co-selection or cross-resistance brought on by other antimicrobials [9–11].

Chloramphenicol is a broad-spectrum antibiotic in the amphenicol group, which inhibits bacterial protein synthesis by binding to 50s ribosomal subunit [12, 13]. One of the most common mechanisms of chloramphenicol resistance is its enzymatic inactivation, particularly by *cat*-encoded acetyltransferases [14]. Another possible resistance mechanisms include the expression of efflux pumps, which frequently function as multidrug extrusion transporters, reduced outer membrane permeability, and target site mutation or alteration. The *cml* and *floR* genes encode specific exporters of chloramphenicol, while the AcrAB-TolC multidrug efflux system can also export chloramphenicol, but to a lesser degree [15]. Several chloramphenicol resistance genes (e.g., *catA*, *catB*, *cmlA* and *floR*) were found to be located on either transposon (e.g., Tn9 and Tn2424) and plasmids. In certain plasmids (e.g., IncX plasmid), chloramphenicol resistance genes were preserved, rather than in others [16]. These genes were found co-located on the same conjugative plasmid with the other AMR genes such as *tet*, *aadA* and *sul*, conferring multidrug resistance phenotype [17]. Despite being banned in food animals, chloramphenicol is used topically to treat eye infections in humans. It has antibiofilm activity, hypothetical low impact on ocular microbiota and narrow resistance rate [18]. There might be the resuscitation of an old antimicrobial medication such as chloramphenicol in the situation that newer medications are not readily available due to the AMR issue. Therefore, investigating the mechanisms behind the persistence of chloramphenicol resistance in the absence of the antibiotic is worthwhile.

To date, Whole Genome Sequencing (WGS) has become an indispensable technique for AMR research and control, with the potential for the discovery of novel antibiotics, identification of AMR in clinical samples, AMR epidemiological surveillance, and tracking of AMR emergence [19]. Information generated by WGS is greatly beneficial for comprehending the origins and transmission of AMR as well as the foundation of AMR mechanisms. This study aimed to determine possible mechanisms associated with the persistence of chloramphenicol resistance in *Escherichia coli* and *Salmonella enterica* isolated from pigs, pork, and humans. WGS was applied to allow genome-wide analysis of CHP-resistant bacteria.

## Materials and methods

### Bacterial isolates and antimicrobial susceptibilities

The *E*. *coli* (n = 106) and *Salmonella* (n = 57) isolates resistant to chloramphenicol (MIC≥ 32 μg/ml) were obtained from our previous AMR monitoring projects during 2007–2008. The *E*. *coli* isolates were obtained from fecal content on rectal swabs of clinically healthy pigs at slaughterhouses. The rectal swab samples were immediately taken from pigs after stunning and bleeding but before the scalding process. *E*. *coli* were isolated and biochemically confirmed [20, 21] and a single colony of *E*. *coli* of each positive sample was collected. Minimum inhibitory concentration (MIC) was determined using agar dilution technique [22]. The isolates exhibited resistance to ampicillin (AMP, 81.2%), chloramphenicol (CHP,100%), ciprofloxacin (CIP, 43.6%), gentamicin (GEN, 47%), streptomycin (STR, 57.3%), sulfamethoxazole (SMX, 67.5%), tetracycline (TET, 98.3%) and trimethoprim (TMP, 91.5%). All the pigs were raised in closed house system in large-scale commercial farming operations with between 11,000 and 13,000 pigs. According to the information provided by farm veterinarians, the antimicrobials routinely used were amoxicillin, chlortetracycline, tylosin, tiamulin, and fosfomycin.

The *Salmonella* isolates were obtained from raw pork (n = 22) at retail markets, and patient’s stools (n = 37) at the hospitals by using ISO 6579:2002 [23] and serotyped using slide agglutination test. One colony of each serovars was collected from each positive sample. The *Salmonella* serovars included Anatum (n = 5), Corvallis (n = 5), Enteritidis (n = 3), Kedougou (n = 17), Newport (n = 2), Panama (n = 4), Rissen (n = 7), Stanley (n = 7), Typhimurium (n = 4) and Weltevreden (n = 3). These *Salmonella* were resistant to AMP (89.5%), CHP (100%), CIP (17.5%), GEN (38.6%), STR (89.5%), SMX (63.2%), TET (89.5%) and TMP (91.5%). All bacterial isolates were stored as 20% glycerol at -80°C.

Each isolate of *E*. *coli* and *Salmonella* carried at least *catA*, *catB*, and *cmlA* but with varying combinations, including *cmlA* only (50.9%, 49%), *catA* only (0, 5.3%), *catB* only (4.7%, 10.5%), *cmlA*/*catA* (0.9%, 12.3%), *cmlA/catB* (28.3%, 10.5%), *catA/catB* (13.2%, 5.3%) and *cmlA/catA/catB* (1.9%, 7.0%).

### Determination of efflux system inhibitor effects on chloramphenicol susceptibility

The MIC value of CHP (Sigma-Aldrich, Saint Louis, MO) was determined in the presence and absence of 25 μg/mL phenylalanine arginine β-naphthylamide (PAβN, Sigma-Aldrich) using two-fold agar dilution method [22]. The concentrations of CHP ranged from 1 μg/mL to 1,024 μg/mL. A 4-fold or more change in the chloramphenicol MIC value following the addition of PaβN was defined as significant. Experiments were repeated on two separate occasions.

### PCR based replicon typing (PBRT)

DNA templates were prepared using whole cell boiling method [24]. All PCR amplification was performed using Top Taq Master Mix kit (QIAGEN, Hilden, Germany) according to the manufacturer’s instruction. All primers used in this study are listed in Table 1. Eighteen replicons including HI1, HI2, I1, X, L/M, N, FIA, FIB, W, Y, P, FIC, A/C, T, FIIAs, F_repB_, K and B/O were PCR amplified using the following thermocycles: one cycle of denaturation at 94°C for 5 min; followed by 30 cycles of denaturation at 94°C for 1 min, annealing at 60°C for 30 s, and extension at 72°C for 1 min; and a final extension at 72°C for 5 min. The exception was for F simplex PCR using F_repB_ primers which employed an annealing temperature of 52°C.

**Table 1:** Primer used in this study.

PCR-reaction	Name	Primer sequences	Amplicon size (bp)	Reference
PBRT				
Multiplex 1	HI1 FW	5’-GGAGCGATGGATTACTTCAGTAC-3’	471	[25]
	HI1 RV	5’-TGCCGTTTCACCTCGTGAGTA-3’		
	HI2 FW	5’-TTTCTCCTGAGTCACCTGTTAACAC-3’	644	
	HI2 RV	5’-GGCTCACTACCGTTGTCATCCT-3’		
	I1 FW	5’-CGAAAGCCGGACGGCAGAA-3’	139	
	I1 RV	5’-TCGTCGTTCCGCCAAGTTCGT-3’		
Multiplex 2	X FW	5’-AACCTTAGAGGCTATTTAAGTTGCTGAT-3’	376	[25]
	X RV	5’-TGAGAGTCAATTTTTATCTCATGTTTTAGC-3’		
	L/M FW	5’-GGATGAAAACTATCAGCATCTGAAG-3’	785	
	L/M RV	5’-CTGCAGGGGCGATTCTTTAGG-3’		
	N FW	5’-GTCTAACGAGCTTACCGAAG-3’	559	
	N RV	5’-GTTTCAACTCTGCCAAGTTC-3’		
Multiplex 3	FIA FW	5’-CCATGCTGGTTCTAGAGAAGGTG-3’	462	[25]
	FIA RV	5’-GTATATCCTTACTGGCTTCCGCAG-3’		
	FIB FW	5’-GGAGTTCTGACACACGATTTTCTG-3’	702	
	FIB RV	5’-CTCCCGTCGCTTCAGGGCATT-3’		
	W FW	5’-CCTAAGAACAACAAAGCCCCCG-3’	242	
	W RV	5’-GGTGCGCGGCATAGAACCGT-3’		
Multiplex 4	Y FW	5’-AATTCAAACAACACTGTGCAGCCTG-3’	765	[25]
	Y RV	5’-GCGAGAATGGACGATTACAAAACTTT-3’		
	P FW	5’-CTATGGCCCTGCAAACGCGCCAGAAA-3’	534	
	P RV	5’-TCACGCGCCAGGGCGCAGCC-3’		
	FIC FW	5’-GTGAACTGGCAGATGAGGAAGG-3’	262	
	FIC RV	5’-TTCTCCTCGTCGCCAAACTAGAT-3’		
Multiplex 5	A/C FW	5’-GAGAACCAAAGACAAAGACCTGGA-3’	465	[25]
	A/C RV	5’-ACGACAAACCTGAATTGCCTCCTT-3’		
	T FW	5’-TTGGCCTGTTTGTGCCTAAACCAT-3’	750	
	T RV	5’-CGTTGATTACACTTAGCTTTGGAC-3’		
	FII_s_ FW	5’-CTGTCGTAAGCTGATGGC-3’	270	
	FII_s_ RV	5’-CTCTGCCACAAACTTCAGC-3’		
Simplex F	F_repB_ FW	5’-TGATCGTTTAAGGAATTTTG-3’	270	[25]
	F_repB_ RV	5’-GAAGATCAGTCACACCATCC-3’		
Simplex K	K/B FW	5’-GCGGTCCGGAAAGCCAGAAAAC-3’	160	[25]
	K RV	5’-TCTTTCACGAGCCCGCCAAA-3’		
Simplex B/O	K/B FW	5’-GCGGTCCGGAAAGCCAGAAAAC-3’	159	[25]
	B/O RV	5’-TCTGCGTTCCGCCAAGTTCGA-3’		
Chloramphenicol resistance genes				
*catA*	catA FW	5’-CCAGACCGTTCAGCTGGATA-3’	454	[26]
	catA RV	5’-CATCAGCACCTTGTCGCCT-3’		
*catB*	catB FW	5’-CGGATTCAGCCTGACCACC-3’	461	[26]
	catB RV	5’-ATACGCGGTCACCTTCCTG-3’		
*cmlA*	cmlA FW	5’-TGGACCGCTATCGGACCG-3’	641	[26]
	cmlA RV	5’-CGCAAGACACTTGGGCTGC-3’		

### Conjugation experiment

Biparental mating was performed to determine co-selection of CHP resistance by other antibiotics. All the CHP-resistant *E*. *coli* (n = 106) and *Salmonella* (n = 57) isolates served as donors. *Salmonella* Enteritidis SE12rif^R^ (CHP MIC = 4 μg/mL) [27] and *E*. *coli* MG1655rif^R^ (CHP MIC = 4 μg/mL) [28] were used as recipients for the *E*. *coli* and *Salmonella* donors, respectively. Neither *E*. *coli* MG1655rif^R^ nor *Salmonella* SE12rif^R^ carry any of the 18 replicons tested. Transconjugants were selected on Luria Bertani agar containing rifampicin (32 μg/mL) and one of the following antibiotics, AMP (150 μg/mL), TET (10 μg/mL) and STR (50 μg/mL). Transconjugants were confirmed to be *E*. *coli* or *Salmonella* by growing on Eosin Methylene Blue agar (EMB; Difco^™^, MI, USA) or Xylose Lysine Deoxycholate agar (XLD; Difco^™^, MI, USA), respectively. The transconjugants were determined for their susceptibilities to CHP and corresponding antibiotics (i.e., AMP, TET or STR) and screened for *catA*, *catB* and *cmlA* [26]. CHP MIC changed by at least four times from the recipients was considered significant. One of transconjugant from each selective pressure plate was selected for further plasmid studying. The *E*. *coli* (n = 11) and *Salmonella* (n = 9) donors and their corresponding transconjugants with CHP MIC ≥4-fold increase (17 *Salmonella* transconjugants and 18 *E*. *coli* transconjugants), were subjected to PBRT.

### Whole genome sequencing (WGS) and bioinformatics analysis

Genomic DNA was extracted from the *E*. *coli* from pigs (n = 3) and *Salmonella* from pork (n = 3) that could transfer CHP resistance genes using ZymoBIOMICS^™^ DNA Miniprep Kit (Zymo Research Corp., Irvine, CA, USA). The degradation of the genomic DNA was assessed by running 5 μL of the DNA on 0.8% agarose gel. The quality and quantity of the genomic DNA were assessed using NanoDrop^™^ 1000 spectrophotometer (Thermo Scientific, Deleware, USA) and submitted for WGS using Oxford Nanopore technologies (ONT) for long read sequencing at Siriraj Long-read Lab, Faculty of Medicine Siriraj Hospital, Mahidol University, Bangkok, Thailand and using Illumina platform Hiseq sequencers (Illumina, San Diego, CA, USA) for short read sequencing at GENEWIZ China and Suzhou Lab, (GENEWIZ, Suzhou, China). Sequencing analysis was performed as previously described [29]. Briefly, adapters were trimmed using Porechop v0.2.4 (https://github.com/rrwick/Porechop). ONT and Illumina reads were quality checked using NanoPlot [30] and FastQC [31], respectively. High quality ONT and Illumina reads were assembled to create hybrid genome using Unicycler [32]. Genomic characteristics including genome size, number of contigs and % GC content were identified using QUAST [33]. Taxonomic identification was performed using Kraken2 [34] and Genome annotation was conducted using NCBI Prokaryotic Genome Annotation Pipeline (PGAP) [35]. The assembled genome/contigs were then analyzed at Center for Genomic Epidemiology website (http://www.genomicepidemiology.org/services/). Multilocus sequence typing (MLST) was performed using MLST 2.0 with the obtained data from http://pubmlst.org. The serotypes of *E*. *coli* were classified by SeroTypeFinder [36]. The *Salmonella* serotypes were confirmed by SeqSero 1.2 [37]. Virulence genes were identified by VirulenceFinder 2.0 [38]. AMR genes were identified by ResFinder4.1 [39]. Mobile genetic elements (MGE) and plasmids were identified by MobileElementFinder v1.0.3 [40] and PlasmidFinder2.1 [41, 42], respectively. Plasmid Multilocus Sequence Typing (pMLST) and replicon sequence type (RST) of IncI1, IncF and IncHI plasmids were identified. Variant calling and core genome alignment with *E*. *coli* LF82 reference strain (accession no. CP082771) and *Salmonella* Typhimurium LT2 (accession no. NC003197.2) were performed by Snippy [43]. Sequences of *E*. *coli* (ST10/2, accession no. SRR12903891 and SRR24437713; ST19/156, accession no. SRR3745274 and SRR25176867) and *Salmonella* (*Salmonella* Weltevreden or ST365, accession no. SRR13853517, SRR24258077 and SRR21734369; and *Salmonella* Rissen or ST469, accession no. SRR13853514 and SRR24401736) with similar serotypes were additionally included to ensure homogeneity. Phylogenetic trees were generated by IQ-TREE [44] and visualized by iTOL v6 [45]. The comparison of genetic environment of CHP resistance genes and the location of insertion sequences (ISs) and mobile genetic elements were achieved using EasyFig [46] and Proksee [47].

### Statistical analysis

The descriptive statistic including percentage was analyzed by excel program. The chi-squared test and z-test using Bonferroni method with SPSS version 22.0 program was used to compare the effect of PAβN on MIC values of chloramphenicol. A *p*-value of <0.05 was considered statistically significant.

## Results

### Effect of efflux pump inhibitor to chloramphenicol MIC

Effects of PAβN on CHP MICs were determined in all *E*. *coli* (n = 106) and *Salmonella* (n = 57) (Table 2). Most *E*. *coli* (67.9%) and *Salmonella* (64.9%) had ≥4-fold CHP MIC decrease in the presence of PAβN. Only in *E*. *coli*, the presence of CHP genes was significantly correlated with the CHP MICs reduction when PAβN was present (*p*<0.05).

**Table 2:** Effect of phenylalanine arginine β-naphthylamide (PAβN) on MIC values of chloramphenicol in *Escherichia coli* (n = 106) and *Salmonella* (n = 57).

Bacterial species	CHP resistance genes pattern	No. of isolates (% within fold) with indicated fold reduction of CHP MIC when presence of PAβN	
		<4 fold	≥4 fold
*Escherichia coli* (n = 106)	*cmlA* (n = 54)	25(73.5)^a^	29(40.3)^b^
	*catB* (n = 5)	2(5.9) ^a^	3(4.2) ^a^
	*cmlA+catA* (n = 1)	1(2.9) ^a^	0 ^a^
	*cmlA+catB* (n = 30)	6(17.6) ^a^	24(33.3) ^a^
	*catA+catB* (n = 14)	0 ^a^	14(19.4) ^b^
	*cmlA+catA+catB* (n = 2)	0 ^a^	2(2.8) ^a^
	Total	34(32.1)	72(67.9)
*Salmonella* (n = 57)	*cmlA* (n = 28)	10(50) ^a^	18(48.7) ^a^
	*catA* (n = 4)	1(5) ^a^	3(8.1) ^a^
	*catB* (n = 6)	2(10) ^a^	4(10.8) ^a^
	*cmlA+catA* (n = 6)	1(5) ^a^	5(13.5) ^a^
	*cmlA+catB* (n = 6)	2(10) ^a^	4(10.8) ^a^
	*catA+catB* (n = 3)	3(15) ^a^	0^b^
	*cmlA+catA+catB* (n = 4)	1(5) ^a^	3(8.1) ^a^
	Total	20(35.1)	37 (64.9)

^a,b^ Values with different superscripts within each row, indicated statistical significantly difference (*p* <0.05) between <4 fold reduction and ≥4 fold reduction of CHP MIC when presence of PAβN.

The presence of *cmlA* was considerably higher in *E*. *coli* with ≥4-fold CHP MIC decrease (29/72, 40.3%) than in those with <4-fold CHP MIC decrease (25/34, 75%). All *E*. *coli* carrying both *catA* and *catB* (14/72, 19.4%) exhibited ≥4-fold CHP MIC decrease in the presence of PAβN (*p*<0.05). Neither of *catA* nor *catB* were detected among *E*. *coli* with <4-fold CHP MIC decrease. In contrast, all the *Salmonella* isolates with <4-fold reduction of CHP MIC in the presence of PAβN (3/20, 15%) carried both *catA* and *catB* genes. None of the *Salmonella* isolates with ≥4-fold CHP MIC reduction contained *catA* and *catB*.

### Co-selection of chloramphenicol resistance by other antibiotics

The *E*. *coli* donors yielded CHP resistant *Salmonella*-transconjugants that exhibited ≥ 4-fold CHP MIC increase when AMP (i.e., E289, E290, E291, E292, E293, E294, E297, E331 and E333), TET (i.e., E290, E291, E292, E293, E294 and E295) and STR (i.e., E329 and E392) were used as selective pressure. Nine *E*. *coli* yielded *Salmonella*-transconjugants with CHP MIC ≥ 32 μg/mL (32–256 μg/mL) in the presence of different selective pressure (AMP, n = 6; TET, n = 6 and STR, n = 1) (Table 3). All the above *E*. *coli* donors could horizontally transfer *cmlA*. *Salmonella* transconjugants carrying *cmlA* with ≥ 4-fold CHP MIC increase (32–128 μg/mL) were obtained when AMP, TET and STR were used as selective pressure.

**Table 3:** Conjugation rates and chloramphenicol resistance phenotype of transconjugants.

Donor	Antibiotic selective pressure	No (%) isolates with CHP resistance transferability	No (%) of transconjugant with ≥4-fold* increase of CHP MIC			
			MIC of CHP of transconjugants		CHP resistance gene	
			<32 μg/mL	≥32 μg/mL	*cmlA*	*catA*
*Escherichia coli* (n = 106)	AMP	9(8.5)	3/9(27.3)	6/9(54.5)	9/9(100)	-
	TET	6(5.7)	-	6/6(100)	6/6(100)	-
	STR	2(1.9)	1/2(50)	1/2(50)	1/2(50)	-
*Salmonella* (n = 57)	AMP	2(3.5)	1/2(50)	1/2(50)	2/2(100)	-
	TET	8(14.0)	-	8/8(100)	7/8(87.5)	1/8(12.5)
	STR	8(14.0)	1/8(5.9)	7/8(87.5)	6/8(75)	1/8(12.5)

AMP, ampicillin; CHP, chloramphenicol; STR, streptomycin; TET, tetracycline.

* When compared to the original CHP MIC of recipients.

Some *Salmonella* produced CHP resistant *E*. *coli*-transconjugants with ≥ 4-fold CHP MIC increase when AMP (i.e., SA449 and SA606), TET (i.e., SA448, SA449, SA461, SA515, SA633, SA639, SA666 and SA759) and STR (i.e., SA448, SA461, SA515, SA633, SA639, SA666, SA741 and SA759) were used as selective pressure. Among these, 10 *Salmonella* donors generated *E*. *coli* transconjugants with CHP MIC ≥ 32 μg/mL (32–512 μg/mL) (Table 3). SA448 and SA741 yielded *cmlA*-carrying *E*. *coli* transconjugants with ≥ 4-fold CHP MIC increase (MIC = 32–256 μg/mL) under the AMP, TET and STR selective pressure. SA448 additionally produced *catA* carrying *E*. *coli* transconjugants exhibiting ≥ 4-fold CHP MIC increase (256–512 μg/mL) in the presence of TET and STR.

### Incompatibility groups of transferable plasmids

Eleven *E*. *coli* that were capable of horizontally transferring CHP resistance carried at least two plasmids (i.e., IncI1/K/F (n = 7), IncI1/F (n = 1), IncHI1/ FIIAs/K (n = 2) and IncHI1/K/FIB / (n = 1)) (Table 4). IncFIIAs plasmids were commonly found in *Salmonella* transconjugants selected by AMP (i.e., AMPE289, AMPE290, AMPE291, AMPE293, AMPE294, AMPE297 and AMPE333) and TET (i.e., TETE290, TETE291, TETE292, TETE293 and TETE295). Two *Salmonella* transconjugants selected by AMP (AMPE331 and AMPE333) and their donors carried IncHI plasmid. Three *Salmonella* transconjugants (i.e., AMPE292, TETE294 and STRE329) acquired *cmlA* from their donors but were not positive to any replicons detected.

**Table 4:** Plasmid of *E. coli* (n = 11) and *Salmonella* (n = 9) donors and corresponded chloramphenicol resistant transconjugants by PBRT.

Donor				Selective pressure	Transconjugant			
ID	Inc	CHP resistance gene	CHP MIC (μg/mL)		ID	Inc	CHP resistance gene	CHP MIC (μg/mL)
E289	I1, K, F	*cmlA*	32	AMP	E289T_AMP	FIIAs	*cmlA*	64
E290	I1, K, F	*cmlA*	64	TET	E290T_TET	FIIAs	*cmlA*	32
				AMP	E290T_AMP	FIIAs	*cmlA*	128
E291	I1, K, F	*cmlA*	32	TET	E291T_TET	FIIAs	*cmlA*	64
				AMP	E291T_AMP	FIIAs	*cmlA*	64
E292	I1, K, F	*cmlA*	32	TET	E292T_TET	FIIAs	*cmlA*	64
				AMP	E292T_AMP	-	*cmlA*	16
E293	I1, K, F	*cmlA*	32	TET	E293T_TET	FIIAs	*cmlA*	64
				AMP	E293T_AMP	FIIAs	*cmlA*	32
E294	I1, K, F	*cmlA*	32	TET	E294T_TET	-	*cmlA*	64
				AMP	E294T_AMP	FIIAs	*cmlA*	64
E295	I1, K, F	*cmlA*	64	TET	E295T_TET	FIIAs	*cmlA*	64
E297	I1, F	*cmlA*	32	AMP	E297T_AMP	FIIAs	*cmlA*	64
E329	HI1, K, FIB, F	*cmlA*	64	STR	E329T_STR	-	*cmlA*	32
E331	HI1, FIIAs, K	*cmlA*	32	AMP	E331T_AMP	HI1	*cmlA*	16
E333	HI1, FIIAs, K	*cmlA*	32	AMP	E333T_AMP	HI1, FIIAs	*cmlA*	16
SA448	HI1, FIIAs	*catA*	256	TET	SA448T_TET	HI1	*catA*	256
				STR	SA448T_STR	HI1, K	*catA*	512
SA449	HI1, FIIAs	*catA*, *cmlA*	128	TET	SA449T_TET	FIB, F	*cmlA*	32
				AMP	SA449T_AMP	FIB, F	*cmlA*	16
SA461	I1, FIB, F	*cmlA*	256	TET	SA461T_TET	F	*cmlA*	32
				STR	SA461T_STR	F	*cmlA*	32
SA515	FIB, A/C, F	*cmlA*	256	TET	SA515T_TET	FIB, A/C, F	*cmlA*	256
				STR	SA515T_STR	FIB, A/C, F	*cmlA*	128
SA606	FIIAs	*catA*, *cmlA*	64	AMP	SA606T_AMP	FIB, FIIAs, F	*cmlA*	32
SA633	FIB, F	*catA*, *catB*, *cmlA*	128	TET	SA633T_TET	FIB	*cmlA*	32
				STR	SA633T_STR	FIB, FIIAs, F	*cmlA*	64
SA639	FIB, F	*catA*, *catB*, *cmlA*	128	TET	SA639T_TET	FIB, F	*cmlA*	32
				STR	SA639T_STR	FIB, FIIAs, F	*cmlA*	32
SA666	FIB, F	*cmlA*	128	TET	SA666T_TET	FIB, F	*cmlA*	32
				STR	SA666T_STR	FIB, F	*cmlA*	16
SA759	FIB, F	*cmlA*	128	TET	SA759T_TET	FIB, F	*cmlA*	32
				STR	SA759T_STR	FIB, F	*cmlA*	32

AMP, ampicillin; CHP, chloramphenicol; STR, streptomycin; TET, tetracycline

The *Salmonella* isolates with the ability to transfer CHP resistance (n = 9) contained at least one plasmid including IncHI1/FIIAs (n = 2), IncI1/FIB/F (n = 1), IncFIB/A/C/F (n = 1), IncFIIAs (n = 1) IncFIB and F (n = 4). Most *E*. *coli* transconjugants (i.e., SA449T_TET, SA449T_AMP, SA515T_TET, SA515T_STR, SA633T_STR, SA639T_TET, SA639T_STR, SA666T_TET, SA666T_STR, SA759T_TET and SA759T_STR) harbored both IncFIB and IncF replicons that were present in their donors. While *E*. *coli* transconjugants of SA488 (i.e., SA448T_TET and SA448T_STR), SA461 (i.e., SA461T_TET and SA461T_STR), SA606 (i.e., SA606T_AMP) and SA633 (i.e., SA633T_TET) acquired only IncHI1, F, FIIAs and FIB plasmids from their respective donors.

### Genomic characteristics of CHP-resistant *E*. *coli* and *Salmonella*

The quality of genome assembly of selected *E*. *coli* and *Salmonella* is shown in Table 5. The genome size and GC content of E290 (serotype O8:H16), E329 (serotype O37:H34), and E333 (serotype O37:H34) was 5,203,479 bp, 50.66%; 5,193,590 bp, 50.46% and 5,193,591 bp, 50.46%, respectively. E290 was made up of 9 contigs including a chromosome and 8 plasmids. E329’s whole genome contained one chromosome and 6 plasmids. Eight contigs, comprising 2 chromosomes and 6 plasmids, were present in E333.

**Table 5:** Quality of whole genome assembly of *Escherichia coli* (n = 3) and *Salmonella enterica* (n = 3).

Bacterial isolate	GC content (%)	Accession No.	Whole Genome length (bp)	Contigs No.	Chromosomal/plasmid contigs size (bp)
E290	50.66	SAMN35027954	5,203,479	1	4,861,935 (Chromosome)
				2–9	100,162 (pO111 plasmid);
					89,517 (IncI1 plasmid);
					70,588 (IncX1 plasmid);
					68,572 (IncFII plasmid); 4,320;
					3,830 (Col440I); 3,003 (Col440I) and 1,552 (Col(MG828))
E329	50.46	SAMN35027955	5,193,590	1	4,849,047 (Chromosome)
				2–7	275,323 (IncFIA(HI1)/HI1B plasmid); 49,549 (IncX1 plasmid);
					9,196 (Col440I);
					4,724; 3,295 and 2,459
E333	50.46	SAMN35027956	5,193,591	1	4,849,048 (Chromosome)
				2 and 4	275,323 (IncFIA(HI1)/HI1B plasmid)
				3, 5–8	49,546 (IncX1 plasmid);
					9,196 (Col440I); 4,724; 3,295 and 2,459
SA448	51.86	SAMN35027957	5,419,175	1	5,101,868 (Chromosome)
				2	232,899 (IncFIA(HI1)/HI1B/Q1 plasmid); 81,966 (IncFII(s) plasmid) and 2,442
SA461	52.05	SAMN35027958	5,127,858	1	4,936,602 (Chromosome)
				2–4	102,883 (IncFII/FIB plasmid);
					83,716 (IncI1 plasmid) and 4,657
SA515	52.06	SAMN35027959	5,214,816	1–8	4,991,361 (Chromosome)
				9–11	114,245 (IncA/C plasmid);
					105,743 (IncFII/FIB plasmid) and 4,657

For *Salmonella*, genome size and GC content in *Salmonella* Weltevreden SA448, *Salmonella* Rissen SA461 and *Salmonella* Rissen SA515 were 5,419,175 bp, 51.86%; 5,127,858 bp, 52.05%, and 5,214,816 bp, 52.06%, respectively (Table 5). SA448 and SA461 comprised 4 contigs, including one for chromosome and 3 for plasmids. There were 11 contigs in SA515 including 3 for plasmids and 8 for chromosomes.

#### Genetic relatedness of CHP-resistant *E*. *coli* and *Salmonella*

**Fig 1:**
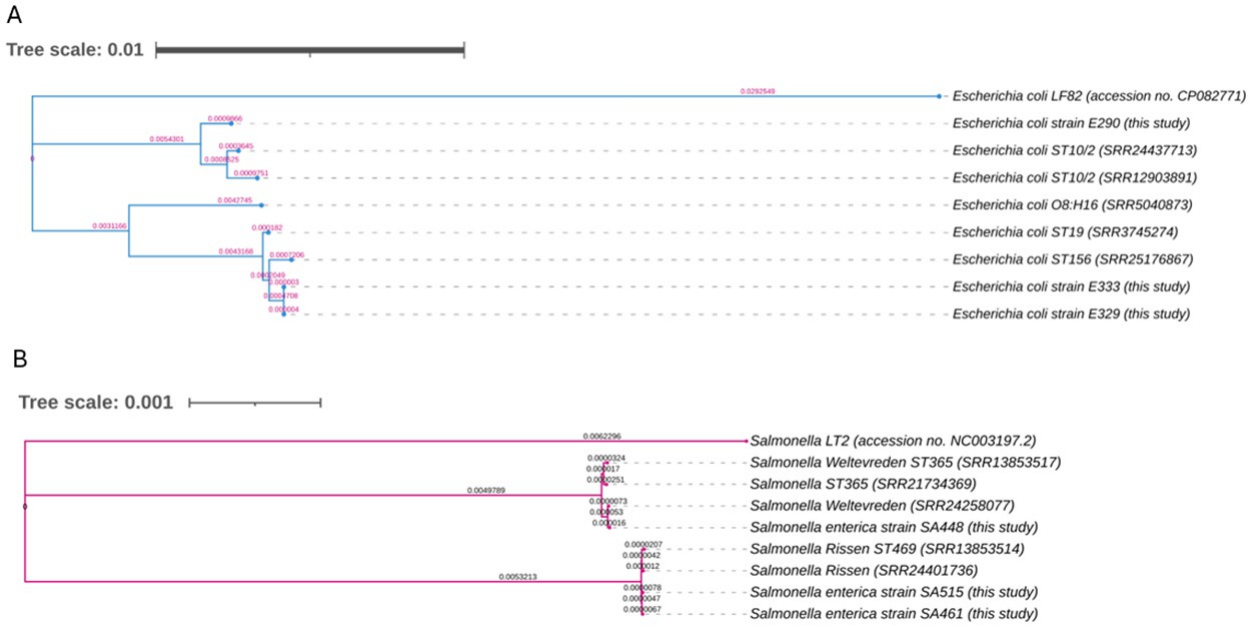
Phylogenetic tree by WGS analysis of *Escherichia coli* (n = 3) and *Salmonella enterica* (n = 3). (A) Chromosomal sequences of (A) E290, E329, E333, reference strains (*E*. *coli* ST10/2; *E*. *coli* O8:H16; *E*. *coli* ST19 and *E*. *coli* ST156) were aligned with *E*. *coli* LF82 and (B) SA448, SA461, SA515, *Salmonella* Weltevreden, *Salmonella* ST365, *Salmonella* Rissen and *Salmonella* ST469 were aligned with *Salmonella* Typhimurium LT2. The single nucleotide polymorphisms (SNPs) were called using Snippy. Phylogenetic trees were generated using Core SNP alignment and visualized by iTOL. The number on the branch indicates genetic changes.

Genome was compared by MLST analysis using Whole Genome Sequence data. Using *E*. *coli* scheme#1, E290, E329, and E333 were identified as ST10, ST156, and ST156, respectively (S1 Table). According to *E*. *coli* scheme#2, they were identified as ST2, ST19, and ST19, respectively (S1 Table). Two distinct clades of *E*. *coli* were identified in phylogenetic trees. E290 was closely related to the *E*. *coli* ST10/2 reference. E329 and E333 were in the same clade with close relationship to *E*. *coli* ST156 (accession no. SRR25176867) and *E*. *coli* O8:H16 (accession no. SRR5040873). SA448, SA461 and SA515 were classified as ST365, ST469 and ST469, respectively. Genetic relatedness was demonstrated using phylogenetic trees (Fig 1B), of which 2 distinct clades were identified. SA448 was closely related to *Salmonella* Weltevreden (accession no. SRR24258077) and *Salmonella* ST365 (accession no. SRR21734369). SA461 and SA515 shared a close lineage with *Salmonella* Rissen (accession no. SRR13853514) and *Salmonella* Rissen ST469 (accession no. SRR13853514).

#### Antimicrobial resistance genes and plasmid characteristics

AMR and virulence genes and plasmids were predicted using whole genome assembly data (Table 6). All three *E*. *coli* carried class1 integrons with *intI1-dfrA12-aadA2-cmlA1-aadA1* gene cassette array, of which *qacL* and *sul3* were present in 3′conserved region. In E290, the class1 integrons were co-localized with *bla*_TEM-1B_ and *tetA* on IncX1 plasmid. The isolates additionally contained pO111 plasmid carrying *bla*_TEM-1B_. The class 1 integrons in E329 and E333 were present on IncFIA(HI1)/HI1B plasmids carrying *aph(3′′)-Ib*, *aac(3)-IId*, *mcr3*.*1*, *mefB*, and *bla*_TEM-1B_. These two isolates additionally possessed IncX1 plasmid with no AMR genes but virulence gene, *mrkA* and chromosomally encoded *tetB*. According to pMLST and RST, IncFII plasmid of the FAB formula F10:A-:B-, and IncI1 plasmid of ST7 were identified in E290 but without AMR genes. IncFIA(HI1)/HI1B plasmids in E329 and E333 were identified as sequence type (ST) 1 for IncHI1and FAB formular F-:A8:B- for IncF.

**Table 6:** Prediction of antimicrobial resistance genes, virulence genes and plasmids from whole genome assembly data in selected *Escherichia coli* (n = 3) and *Salmonella enterica* (n = 3).

Bacterial species	Strain	Serotype	Sample source	CHP MIC (μg/mL)	MLST	Gene mutation	Contig No.	Plasmid		Acquire antimicrobial resistance genes	Virulence genes
								Inc group	pMLST / FAB formula		
*Escherichia coli*	E290	O8:H16	Pig	64	10^a^/2^b^	• *parC*:p.E62K, • *gyrB*:p.A306S	1^c^	-	-	*-*	*yehD*, *terC*, *fimH*, *hlyE*, *iss*, *AslA*, *csgA*, *fdeC*, *fyuA*, *hha*, *fimH*, *yehB*, *gad*, *yehC*, *nlpl*, *astA*, *irp2*, *yehA*
							2	pO111	-	*bla*_TEM-1B_	*-*
							3	I1	7	*-*	*-*
							4	X1	-	*aadA1*, *aadA2*, *dfrA12*, *sul3*, *tetA*, *tetM*, *bla*_TEM-1B_, *qacL*, *cmlA*	*-*
							5	FII	F10:A-:B-	*-*	*traT*, *sepA*
	E329	O37:H34	Pig	64	156^a^/19^b^	• *gyrA*:p.S83L*, *gyrA*:p.D87H*, • *parC*:p.S80I*, *parC*:p.E62K, • *ampC*-promoter:g.-18G>A, *ampC*-promoter:g.-1C>T, • *pmrB*:p.Y358N, *pmrB*:p.D283G, • *pmrA*:p.G144S,	1^c^	-	-	*tetB*	*hra*, *gad*, *hha*, *fimH*, *terC*, *hlyE*, *yehC*, *csgA*, *yehB*, *nlpl*, *fdeC*, *lpfA*, *yehA*, *yehD*, *iss*,
							2	HI1A, HI1B(R27) and FIA(HI1)	1(HI1)/F-:A8:B-	*aadA1*, *aadA2*, *aph(3”)-Ib*, *aac(3)-IId*, *mcr3*.*1*, *sul3*, *dfrA12*, *tetM*, *blaTEM-1B*, *mefB*, *qacL*, *cmlA1*	*-*
							3	X1	-	*-*	*mrkA*
	E333	O37:H34	Pig	512	156^a^/19^b^	• *gyrA*:p.S83L*, *gyrA*:p.D87H*, • *parC*:p.S80I*, *parC*:p.E62K, • *ampC*-promoter:g.-18G>A, *ampC*-promoter:g.-1C>T, • *pmrB*:p.D283G, *pmrB*:p.Y358N, • *pmrA*:p.G144S	1^c^	-	-	*tetB*	*nlpl*, *fdeC*, *iss*, *yehB*, *lpfA*, *yehC*, *gad*, *terC*, *hha*, *hra*, *yehA*, *hlyE*, *csgA*, *fimH*, *yehD*
							2&4	HI1A,HI1B (R27), FIA(HI1)	1 (HI1)/F-:A8:B-	*aac(3)-IId*, *aadA1*, *aph(3”)-Ib*, *aadA2*, *mcr3*.*1*, *sul3*, *dfrA12*, *tetM*, *bla*_TEM-1B_, *mefB*, *qacL*, *cmlA1*	
							3	X1	-	*-*	*mrkA*
*Salmonella enterica*	SA448	Weltevreden	Pork	256	365	• *acrB*:p.L40P, *acrB*:p.F28L, • *parC*:p.T57S*	1^c^	-	-	*aac(6’)-Iaa*	*nlpl*
							2	HI1A, HI1B (R27), FIA(HI1), Q1	2 (HI1)/F-:A8:B-	*aph(6)-Id*, *aph(3”)-Ib*, *sul2*, *tetB*, *catA1*	*-*
							3	FII(s)	S1:A-:B-	*-*	*-*
	SA461	Rissen	Pork	256	469	• *parC*:p.T57S*, • *acrB*:p.L40P, *acrB*:p.F28L	1^c^	-	-	*aac(6’)-Iaa*, *tetA*	*nlpl*
							2	FII/FIB	F46:A-:B-	*aadA2*, *aph(3’)-Ia*, *aadA1*, *dfrA12*, *sul3*, *bla*_TEM-1B_, *mefB*, *qacL*, *cmlA1*	*traT*, *traJ*, *anr*
							3	I1	113^d^ and 115^d^	*-*	*-*
	SA515	Rissen	Pork	256	469	• *parC*:p.T57S*, • *acrB*:p.L40P, *acrB*:p.F28L	1, 3, 5, 7–14^c^	-	-	*aac(6’)-Iaa*, *tetA*	*nlpl*
							2	A/C	3	*aph(6)-Id*, *aph(3”)-Ib*, *sul2*, *blaCMY-2*, *floR*	*-*
							4	FII/FIB	F46:A-:B-	*aadA2*, *aph(3’)-Ia*, *aadA1*, *dfrA12*, *sul3*, *tetA*, *blaTEM-1B*, *mefB*, *qacL*, *cmlA1*	*traT*, *traJ*, *anr*

^a^ MLST results which were typed using MLST *Escherichia coli* scheme #1

^b^ MLST results which were typed using MLST *Escherichia coli* scheme #2

^c^ chromosomal DNA contig

^d^ Nearest results that obtained from pMLST 2.0

CHP, chloramphenicol; MIC, minimum inhibitory concentration; MLST, muti locus sequence typing.

All *Salmonella* isolates carried at least one CHP resistance genes including *catA* in SA448 and *cmlA1* in SA461 and SA515 (Table 6). Class1 integrons with *cmlA1* on IncFII/FIB plasmids was found in both SA461 and SA515 but not SA448. The *aadA2*, *aph(3’)-Ia*, *aadA1*, *sul3*, *dfrA12*, *bla*_TEM-1B_, *mefB* and *qacL* genes were co-localized on the same plasmid. SA515 additionally contained IncA/C plasmid with *floR*, as well as *aph(6)-Id*, *aph(3”)-Ib*, *sul2* and *bla*_CMY-2_. IncFIA(HI1)/HI1B/Q1 plasmid of SA448 carried *catA* as well as *aph(6)-Id*, *aph(3”)-Ib*, *sul2*, and *tetB*. In addition, *acc(6’)-Iaa* was chromosomally encoded in all three *Salmonella* isolates. The *tetA* gene was exclusively found in the chromosome of SA461 and SA515.

Based on pMLST and RST results (S2 Table), IncFIA(HI1)/HI1B/Q1 plasmids in SA448 were identified as ST2 and F-:A8:B-. In the same isolate, IncFII(s) plasmid belonged to S1:A-:B- FAB formula was found with the absence of AMR genes. IncFII/FIB plasmids carrying *cmlA1* in SA461 and SA515 was in F46:A-:B- FAB formula. SA461 additionally carried IncI1plasmid without resistance and virulence genes. IncA/C plasmid of ST3 lacking *floR* was found in SA515.

#### Structural comparison of plasmid carrying *cmlA1*, *catA* and *floR*

**Fig 2:**
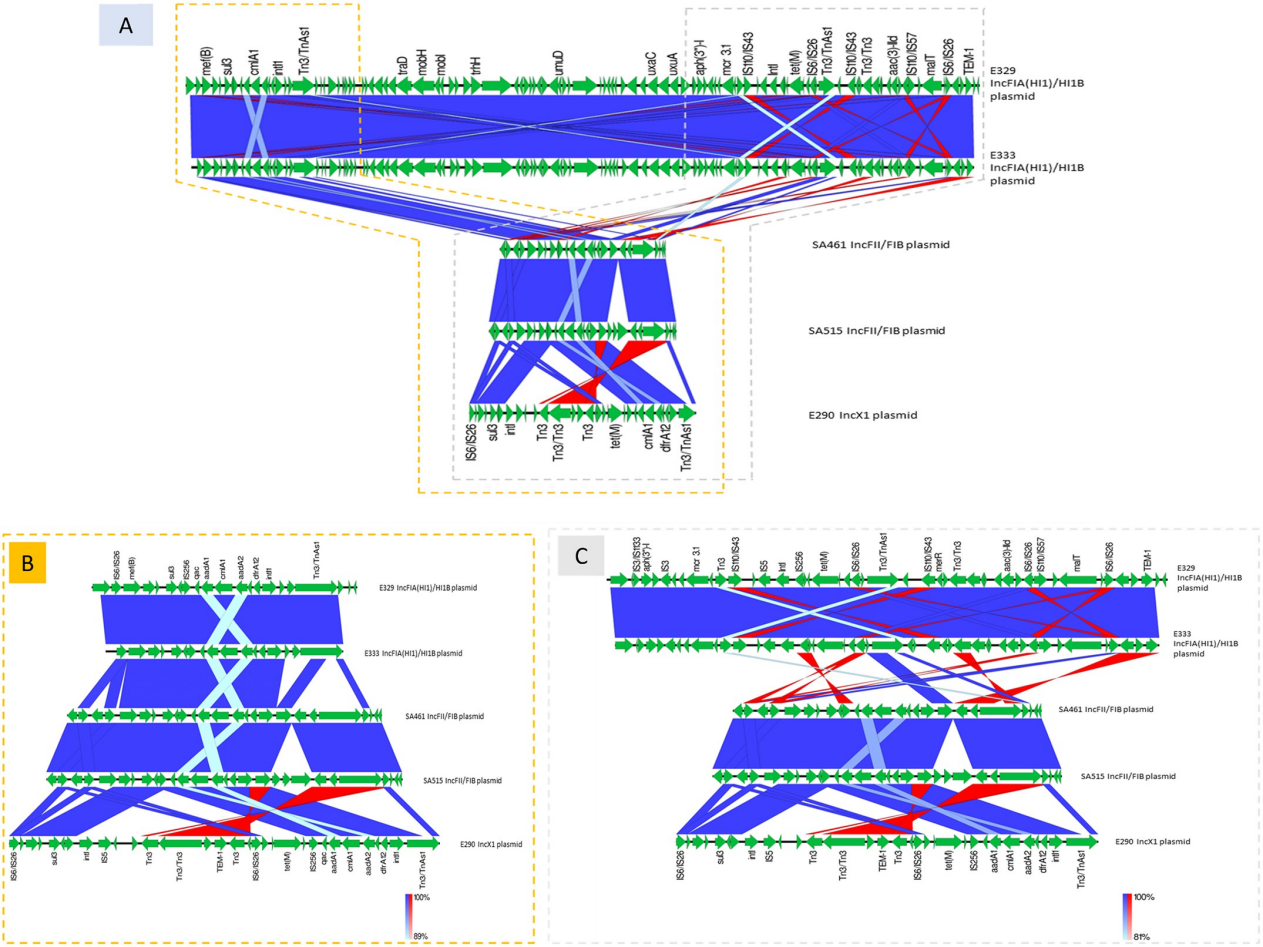
Alignment of plasmids carrying *cmlA1* of *E*. *coli* (n = 3) and *Salmonella enterica* (n = 2). (A) Whole plasmid sequences of IncFIA(HI1)/HI1B plasmid from E329 and E333, IncFII/FIB plasmid from SA461, SA515, and IncX1 plasmid from E290 were compared. Green arrows indicate the position and direction of genes. Blue vertical blocks indicate regions of shared similarity shaded according to BLASTn (dark blue for matches in the same direction and red for inverted matches). (B) A zoomed-in view (yellow dashed line-box) shows the area containing class1 integrons with *cmlA1* gene cassette, which is flanked by Tn*3*-like element *Tn3* or Tn*As1* family transposase at upstream and IS*6*-like element of IS*26* family transposase at downstream. (C) A zoomed-in view (gray dashed line-box) shows the position of *bla*_TEM-1B_ and IS*6*-like element of IS*26* family transposase that are located downstream of class1 integrons in IncFIA(HI1)/HI1B plasmid of E329 and E333 and IncX1 plasmid of E290 and upstream in IncFII/FIB plasmid from SA461 and SA515.

IncFIA(HI1)/HI1B plasmid from E329 and E333 and IncFII/FIB plasmid from SA461 and SA515 shared similar structure and sequences (Fig 2). AMR genes and ISs in two regions located upstream and downstream of IncFIA(HI1)/HI1B plasmids are distributed in IncFII/FIB and IncX1 plasmid. In the upstream region, class1 integrons with *dfrA12*-*aadA2-cmlA1*-*aadA1* gene cassette array and *qacL*-IS*256-sul3* conserved region were identified in all plasmids. Class 1 integrons were flanked by Tn*3*-like element *Tn3* or Tn*As1* family transposase at upstream and IS*6*-like element of IS*26* family transposase at downstream. The downstream region with *bla*_TEM-1B_ and ISs/transposons (i.e., IS*6*-like element of IS*26* family transposase, IS*256* and *Tn3*) were identified in all plasmids.

**Fig 3:**
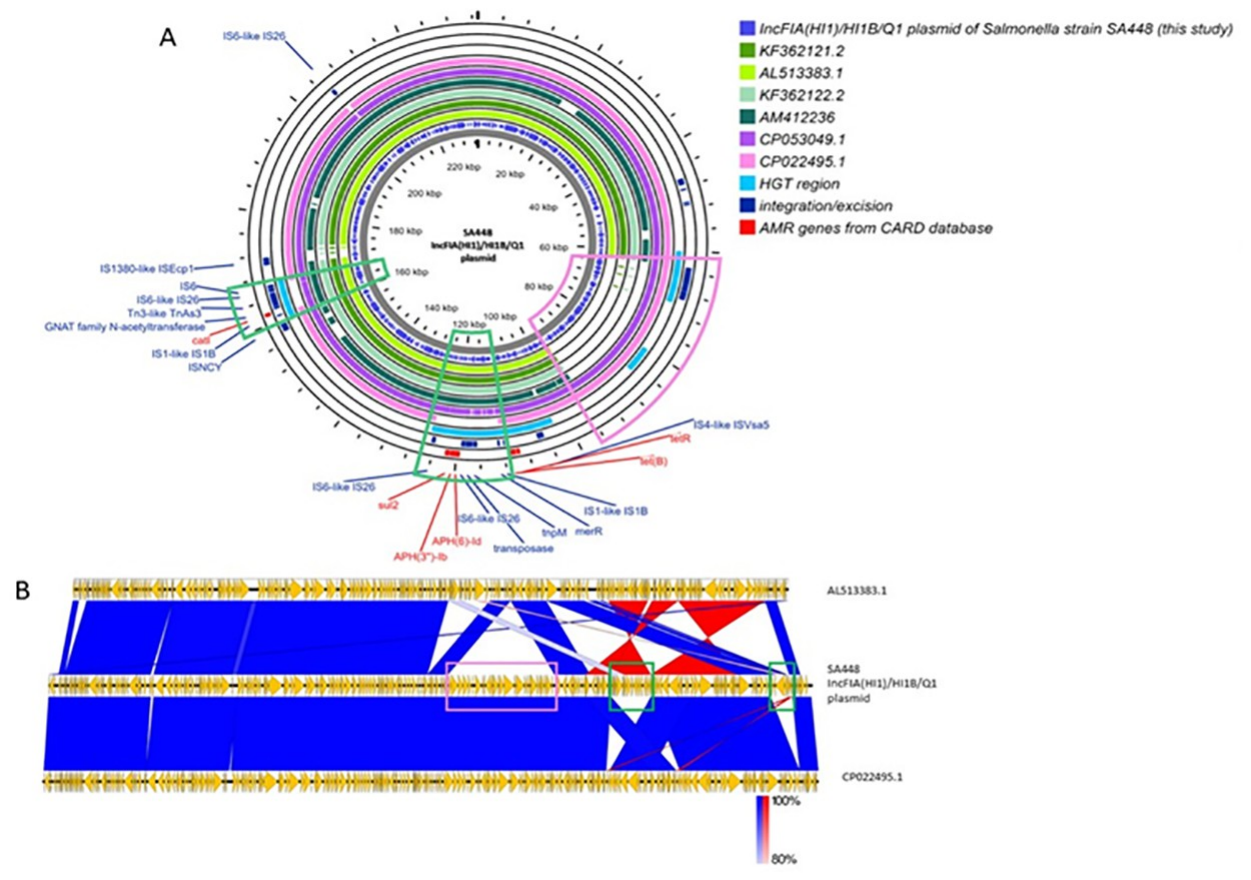
Circular comparison of *catA* carrying IncFIA(HI1)/HI1B/Q1 plasmid of SA448. (A) The comparison was against the highest similarity sequence obtained from NCBI database (accession no., AL513383.1, KF362121.2, KF362122.2, AM412236, CP053049.1 and CP022495.1). The outer red circle indicates AMR genes from CARD database. Green, pink and purple color are sequences of the high degree similarity of reference plasmids. The light blue and navy blue denote the location of horizontal gene transfer (HGT) region and integration/excision genes, respectively. Green block arcs show the area containing AMR genes found in among all plasmids, except for CP022495.1. The pink block arc indicates the area containing HGT region and integration/excision region that are found only in IncFIA(HI1)/HI1B/Q1 plasmid of SA448, CP053049.1 and CP022495.1. (B) The whole plasmid sequence comparison of IncFIA(HI1)/HI1B/Q1, AL513383.1 and CP022495.1. Yellow arrows indicate the position and direction of the genes. Vertical blocks between sequences indicate regions of shared similarity shaded according to BLASTn (dark blue for matches in the same direction or red for inverted matches). Green and pink rectangles correspond to green and pink block mentioned above.

IncFIA(HI1)/HI1B/Q1 plasmid from SA448 had the highest sequence similarity with p30155-1 plasmid from *Salmonella* Derby originated from swine (accession no., CP053049.1) (Fig 3). The *tetB*, *aph(6)-Id*, *aph(3”)-Ib*, *sul2* and *catA1* genes were found in all plasmids, except CP022495.1. Horizontal gene transfer (HGT) regions were present only in IncFIA(HI1)/HI1B/Q1 plasmid of SA448, CP053049.1 and CP022495.1 (Fig 3A and 3B). The *catA1* gene was in the HGT region and flanked by IS*1*-like element IS*1B* family transposase and Tn*3*-like element Tn*As3* family transposase.

**Fig 4:**
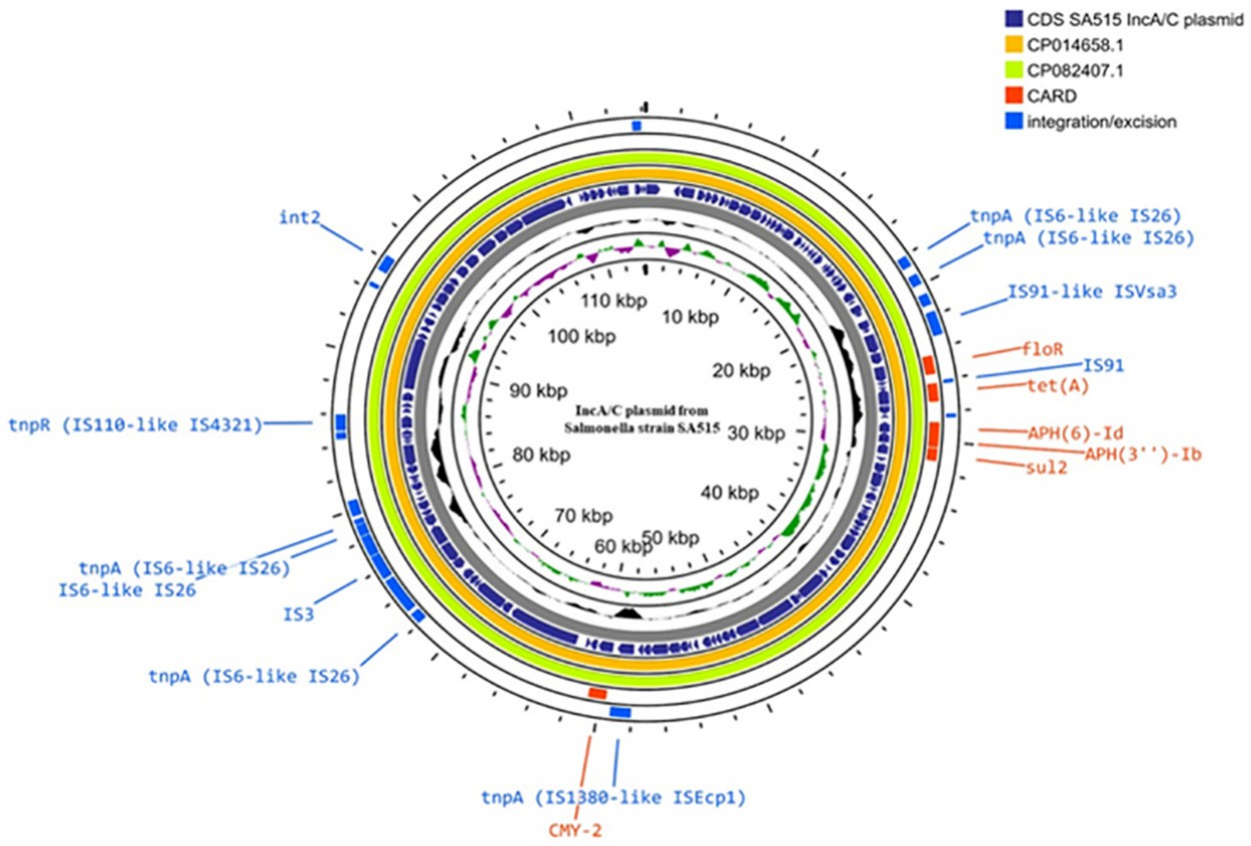
Circular comparison of *floR*-carrying IncA/C plasmid of SA515. The comparison was made to the sequence with the highest similarity obtained from NCBI database (pSANI-1736, accession no., CP014658.1 and pF18S036-1, CP082407.1). Blue and red circles indicate the integration/excision genes and AMR genes from CARD database, respectively. The aligned sequences in yellow and green circles show a significant degree of similarity of IncFIA(HI1)/HI1B/Q1 plasmid to pSANI-1736 from bovine-*Salmonella* Anatum and pF18S036-1 from swine-*Salmonella* Ohio.

IncA/C plasmid carrying *floR* identified in *Salmonella* strain SA515 was closely related to pSANI-1736 from *Salmonella* Anatum isolated from bovine (accession no., CP014658.1) and pF18S036-1 from *Salmonella* Ohio isolated from swine (accession no., CP082407.1) (Fig 4). The common resistance genes found in all plasmids included *tetA*, *aph(6)-Id*, *aph(3”)-Ib*, *sul2* and *bla*_CMY-2_. The *floR* gene was flanked by IS*91*-like element IS*Vsa3* family transposase and IS*91* family transposase. The *int2* gene of class 2 integrons were additionally identified.

## Discussion

It is becoming more well recognized that bacteria can continue to develop resistance even when antibiotics are banned, of which CHP resistance is among the most well-known examples of this phenomena. Our findings demonstrated that the mechanisms involved in the persistence of CHP resistance in *E*. *coli* and *Salmonella*, including cross-resistance mediated by the expression of multidrug efflux systems using proton motif force and co-selection of R plasmids by other antimicrobial agents.

All *E*. *coli* and *Salmonella* in this study were resistant to CHP, of which most *E*. *coli* (67.9%) and *Salmonella* (56.1%) exhibited ≥4-fold CHP MIC decrease when PAβN is present, indicating the involvement of the RND multidrug efflux systems in their CHP resistance phenotype. Hence, any antimicrobial agent that activates the expression of a multidrug efflux pump (s) may promote cross resistance to CHP. At the same time, most CHP-resistant *E*. *coli* and *Salmonella* with <4-fold reduction of CHP MICs (0 to ≤ 2 folds) in the presence of PAβN, carried *cmlA* encoding a proton motif force dependent multidrug efflux pump that belongs to Major Facilitator superfamily. This implies that the CmlA multidrug efflux pump confers a low-level resistance to CHP.

A plasmid borne- or chromosomally encoded- *cat* gene encodes chloramphenicol acetyltransferase (CAT) enzyme. The genes were common in the CHP-resistant *E*. *coli* and *Salmonella* (41% and 51%, respectively), in agreement with previous studies [48, 49]. The contribution of RND efflux pumps in CHP resistance was limited in the CHP-resistant *Salmonella* carrying *catA* and *catB* (<4-fold CHP MIC reduction). In contrast, the presence of PAβN reduced CHP MIC ≥4 fold in *E*. *coli* carrying *catA* and *catB*, indicating the accumulative effects of enzymatic and non-enzymatic mechanisms on CHP resistance.

In conjugation experiment, AMP, TET and STR selective pressure co-selected for CHP resistance in *E*. *coli* or *Salmonella* transconjugants, similar to previous studies conducted in *E*. *coli* isolates from humans and food animals from United States [50]. Almost all CHP-resistant *E*. *coli* or *Salmonella* transconjugants carried *cmlA*, while one CHP-resistant *Salmonella* transconjugants contained *cat*, indicating the localization of *cmlA* and *cat* on transferable R plasmids and in agreement with previous studies [51, 52]. However, the CHP MIC among *Salmonella* and *E*. *coli* transconjugants increased ≥ 4 folds (from 4 to 32 and 4 to 128 folds, respectively) and was inconsistent with the limited contribution of *cmlA* suggested by the PAβN experiment. It was possible that the contribution of *cmlA* to CHP resistance level was more clearly observed in *in vitro* settings where the recipients with low CHP MIC were used (CHP MIC of 4 μg/ml for both *E*. *coli* MG1655rif^r^ and *Salmonella* Enteritidis SE12 rif^r^).

In the PBRT experiment, no plasmids were detected in some *cmlA* carrying *Salmonella* transconjugants (E292T_AMP, E294T_TET and E329T_STR), in agreement with their corresponded donors. The possibility exists that the gene was located on plasmids that were not part of the PBRT scheme used in this study. It was previously suggested that PBRT may be unable to detect some replicons on the large multiple-replicon plasmids due to mutation through transpositional alterations and unknown existence of the new replicons [53]. It highlighted the necessity of expanding the PBRT to enable rapid plasmid screening. IncF plasmids were predominantly found in *cmlA* carrying transconjugants, in agreement with a previous study [52]. This was not surprising because IncF plasmids are prevalent in Enterobacterales. In addition to IncF plasmids, IncHI1 and IncA/C plasmids were horizontally transferred (E331T_AMP, E333T_AMP, SA448T_TET, SA448T_STR, SA515T_TET, SA515T_STR) in agreement with a previous study conducted in *Salmonella* clinical isolates [52, 54]. Nevertheless, it remained unclear which plasmid carried *cmlA* and this could be a subject of future study.

According to WGS analysis, *cmlA* was identified on class1 integrons with *dfrA12*-*aadA2*-*cmlA1-aadA1*cassette array either located on IncX1 plasmid (in E290), in agreement with previous studies conducted in *E*. *coli* from human and livestock [55, 56] or IncFIA(HI1)/HI1B (in E329 and E333). In SA461 and SA515 from pork, *cmlA* was found on IncFII/FIB plasmid carrying class 1 integrons carrying *dfrA12*-*aadA2*-*cmlA1-aadA1* gene cassette array, in agreement with previous reports in Europe [57]. The gene was additionally found located on class 1 integrons with *estX-psp-aadA2-cmlA1-aadA1* cassette array in *E*. *coli* and *Shigella* from China and Taiwan [58, 59]. Besides, the *cmlA* gene was previously found on other transferable plasmids such as IncF, IncA/C, IncHI1 and IncR plasmids [52, 60]. As such, co-selection for *cmlA1* conferring CHP resistance could occur when trimethoprim, streptomycin, and spectinomycin are used. The presence of *cmlA* on different class 1 integrons located on different plasmids could explain its widespread along the food chain.

As evidenced by a comparison of structural variations in WGS, several resistance genes resided on *cmlA* or *catA*-carrying plasmids. All plasmids (IncFIA(HI1)/HI1B, IncFII/FIB and IncX1 plasmids) carried class 1 integrons with *dfrA12*-*aadA2-cmlA1*-*aadA1* cassette array. IncFII/FIB of SA461 and SA515 contained the same upstream and downstream genes as IncFIA(HI1)/HI1B of E329 and E333, but in a different order. In addition, the *aph(3”)-Ib*, *mcr3*.*1*, *tetM*, *aac(3)-IId* and *bla*_TEM-1_ genes were found at the downstream of IncFIA(HI1)/HI1B in E329 and E333. Notably, *cmlA* and *bla*_TEM-1_ were found together in all plasmids, similar to previous study [61]. The class 1 integrons with *dfrA12-aadA2-cmlA1-aadA1* cassette array were flanked by Tn*As1*/ Tn*3* at upstream and IS*6*/ IS*26* at downstream, in agreement with previous studies [62, 63]. Tn*As1* and Tn*As3* are members of the Tn*21* family with Tn *As3* more frequently linked to *cmlA1* and *intI1* [64]. The mobilization of phenicol resistance genes is commonly mediated with transposons, particularly Tn*21* [64], in agreement with the observation in this study. Flanking by insertion sequences facilitates the mobilization of many genes, whereas IS26 and other IS*6* family members was previously shown to enhance the ability for replicon fusions, or the cointegration of donor and target replicons, contributing to the spread of the resistance determinants [65]. Another evidence is that SA448 carried *catA* that was situated between IS*1B* and Tn*As3* on IncFIA(HI1)/HI1B/Q1 plasmid, similar to a previous study [66]. Besides, GCN5-related N-acetyltransferase (GNAT) encoding gene that is primarily implicated in resistance to CHP, aminoglycosides and streptogramins was inserted between *catA* and Tn*As3* immediately upstream to IS*6*/IS*26* [67]. GNAT and a global regulator, *clp*, were linked and co-transcribed for CHP detoxification leading to CHP resistance. Taken together, these findings are evidence of how the co-selection of CHP resistance is mediated by other antibiotics.

According to the structural comparison, the nearly all sequences of the IncFIA(HI1)/HI1B/Q1 plasmid of SA448 and the reference plasmid (accession no. CP022495.1) were identical with the exception of two horizontal gene transfer regions (HGT region) containing IS*1B*-*catA*-GNAT-Tn*As3*-IS*26*-IS*6* and IS*26*-*aph(6)-ld*-*aph(3”)-lb*-*sul2*-IS*26*. The CP022495.1 reference plasmid originated from *Salmonella* Derby; however, further details regarding origins and locations were absent. Regardless, the findings demonstrated the dynamics of AMR gene mobilization through R plasmids, contributing to AMR dissemination.

CHP is a substrate for AcrAB-TolC multidrug efflux pump. In this study, SA448, SA461 and SA515 carried point mutations in chromosomally encoded *acrB*, leading to amino L40P and F28L acid substitution of AcrB. The relation of the amino acid substitution to any specific AMR was not predicted due to a lack of the mutation available in the ResFinder database. The mutations suggested that the CHP resistance phenotype in the isolates was not attributed to the expression of this system.

There are limitations to this study that should be noted. Only PaβN that primarily reduces the efflux effect of RND efflux pumps was used, therefore, the effects of efflux pumps in different families were disregarded. Although WGS produces a massive amount of data, a lot of it could be irrelevant or ambiguous. Despite advances in genomics, many resistance genes are still not identified and not comprised in databases available for AMR analysis. In addition, the limited number of CHP-resistant isolates from pigs were included. Increasing the number of isolates from other livestock would reveal more insights of the mechanisms contributing to the CHP resistance persistence.

In conclusion, the results unveiled cross resistance by multidrug efflux system and co-selection of R plasmids by other antimicrobial drugs as key mechanisms, contributing to the persistence of CHP resistance in *E*. *coli* and *Salmonella* in this study. Reduction and optimization of antimicrobial consumption is necessary to combat AMR. It is evident that restricting the use of a single antimicrobial agent is insufficient to address the issue. In addition to the prohibition on antibiotic usage, several policies and initiatives that reduce the need for antibiotics and delay the spread of AMR are necessary e.g., farm biosecurity, infection control, vaccination program, prudent antimicrobial use etc. Veterinarians are advised to undertake antimicrobial stewardship to improve antimicrobial utilization and decrease the indiscriminate use of antibiotics. Laboratory-based AMR surveillance should be prioritized and conducted at phenotypic and genotypic level with coordination among sectors.

## Supporting information

S1 Table Allele types of *Escherichia coli* and *Salmonella*. (PDF)

S2 Table Allele types of IncA/C, IncHI1, IncI1 and IncF plasmid. (PDF)
