# Supplementary material for: Molecular basis of the persistence of chloramphenicol resistance among Escherichia coli and Salmonella spp. from pigs, pork and humans in Thailand
Source: PLoS One. 2024 May 24;19(5):e0304250. doi: 10.1371/journal.pone.0304250 (PMC11125496; doi:10.1371/journal.pone.0304250)
Supplement: S1 Table — (PDF) [file pone.0304250.s001.pdf]

Table S1A. Allele types of *Escherichia coli* were detected by MLST *Escherichia coli* scheme1

Isolate	Sequence Type	Allele on locus						
		adk	fumC	gyrB	icd	mdh	purA	recA
E290	10	10	11	4	8	8	8	2
E329	156	6	29	32	16	11	8	44
E333	156	6	29	32	16	11	8	44

Table S1B. Allele types of *Escherichia coli* were detected by MLST *Escherichia coli* scheme2

Isolate	Sequence Type	Locus							
		dinB	icdA	pabB	polB	putP	trpA	trpB	uidA
E290	2	8	2	7	3	7	1	4	2
E329	19	7	37	4	10	26	7	4	2
E333	19	7	37	4	10	26	7	4	2

Table S1C. Allele types of *Salmonella* were detected by MLST scheme

Isolate	MLST profile	Sequence Type	Locus						
			aroC	dnaN	hemD	hisD	purE	sucA	thrA
SA448	*senterica*	365	130	97	25	125	84	9	101
SA461	*senterica*	469	92	107	79	156	64	151	87
SA515	*senterica*	469	92	107	79	156	64	151	87
