# Supplementary material for: Molecular basis of the persistence of chloramphenicol resistance among Escherichia coli and Salmonella spp. from pigs, pork and humans in Thailand
Source: PLoS One. 2024 May 24;19(5):e0304250. doi: 10.1371/journal.pone.0304250 (PMC11125496; doi:10.1371/journal.pone.0304250)
Supplement: S2 Table — (PDF) [file pone.0304250.s002.pdf]

Table S2A. Allele types of IncHI1 plasmid

isolate	contig No.	Plasmid incompatibility	pMLST	locus					
				HCM1_043	HCM1_064	HCM1_099	HCM1_116	HCM1_178ac	HCM1_259
E329	2	HI1	1	1	1	1	1	1	1
E333	2	HI1	1	1	1	1	1	1	1
SA448	2	HI1	2	1	2	1	1	1	1

Table S2B. Allele types of IncA/C plasmid

isolate	contig No.	Plasmid incompatibility	pMLST	locus			
				repA	parA	parB	A053
SA515	2	A/C2	3	2	2	2	1

Table S2C. Allele types of IncI1 plasmid

isolate	contig No.	Plasmid incompatibility	pMLST	locus					Clonal complex
				ardA	pilL	repI1	sogS	trbA	
E290	3	I1	7	1	2	2	4	5	CC-7

Table S2D. Allele types of IncF plasmid

isolate	contig No.	Plasmid incompatibility	RST	locus						
				FIA	FIB	FIC	FII	FIIK	FIIS	FIIY
E290	5	F	F10:-:-	-	-	-	10	-	-	-
SA461	2	F	F46:-:-	-	-	-	46	-	-	-
SA515	4	F	F46:-:-	-	-	-	46	-	-	-
